# A system dynamics-based synergistic model of urban production-living-ecological systems: An analytical framework and case study

**DOI:** 10.1371/journal.pone.0293207

**Published:** 2023-10-19

**Authors:** Jiawei Wu, Junlin Huang

**Affiliations:** School of Geographical Sciences, Hunan Normal University, Changsha, China; Sichuan Agricultural University, CHINA

## Abstract

Human-land coordination represents urbanization and is a key component of urban modernization. In this study, the theory of system dynamics was introduced, in which a "production-living-ecological" complex system was used based on the human-land coordination concept. Moreover, the characteristics of system dynamics of causal cycle, dynamic and sustainable development, man-land synergy, integrity and openness, and self-organization and adaptability were analyzed by dividing it into three subsystems: urban production, urban living, and urban ecological subsystems. Here, causal feedback and system structure flow diagrams were designed using causal loop diagrams and system structure flow diagrams to evaluate the causal relationships between variables and quantitatively analyzing their interactions between variables and predicting the future development of variables. Changsha City, China was selected as the case study area, where we constructed system dynamics practice equation model was then constructed to determine the interaction between the subsystems. Our findings indicate that by the year 2035 in the future, the overall trend of factors influencing the function of the subsystems such as population, GDP and built-up area are positively correlated with an increasing trend, and there are interactions between. Furthermore, these factors interact with each other, and a mutual correlation was found among the production-living-ecological functions system, Therefore, this study provides a novel perspective and exploratory practice for the study of the synergistic coupling of ecological, production, and living functions of cities and evaluating high-quality development of cities. Thus, the coupling and coordination of urban production, living and ecological functions reflects the coupling and coordination of the "people-land" relationship, which is the key to high-quality urban development.

## Introduction

In 2022, the Chinese government proposed a people- centered approach to urbanization, which incorporates a regional economic and territorial space structure to support high-quality development. Urbanization promotes coordinated regional development and fosters an improved quality of life for the people. It involves the spatial expansion of cities and towns and modernization of systems and cultures [1–3]. Hence, the "human-land" relationship is considered as an essential tool to identify approaches to high-quality development of urban areas [4–7]. The human-land relation demonstrates a complementary relationship between human economic activities and environmental resource capacity [4,8,9]. Furthermore, in terms of urban function, it reflects the degree of rationalization and efficiency of "production, living and ecology" functions in the city [10,11]. By understanding the coordinated developments between the subsystems of the "production-living-ecological", proper urban development can be achieved [12–14]. However, for a long time, the accelerated process of urbanization has put serious pressure on the ecological space of cities and human production activities, such as the uncontrolled expansion of construction land, have continuously caused negative impacts on the ecological environment. This has resulted in the trade-offs and incoherence between the "production-life-ecology" functions of cities, and has attracted the attention of many scholars [15,16].

Since 2012, research on the "production-living-ecological" system has gained attention. The concept includes three primary aspects: The first is the definition of the connotation of the "production-living-ecological" functions and the construction of the index system and the evaluation based on the index system [17–20]. The second is to analyze the relationship between the coordination characteristics of the "production-living-ecological" functions and land planning [21]. Scholars such as Zhang Z, Shan Y, and Ni W quantitatively analyzed the process of mutual influence of transformation and coupling coordination among the "production-living-ecological" functions and proposed the theory that production functions determine living functions and living functions influence ecological functions. For example, the alpine grassland on the Qinghai-Tibet Plateau need to realize the coordinated development of ecological, production, and living functions of the alpine grassland ecosystem by regulating the population carrying capacity according to the mutual influence mechanism and reasonable proportional structure of the production-living-ecological functions [22–25]. Lastly, identifying and analyzing the "production-living-ecological" space using the land functions perspective [26]. For instance, Fu [27]and Heng et al [28,29]. used the "production-living-ecological" and found out that there was a poor overall spatial arrangement, in which spatial functions were not complementary and integrated.

Several studies like those of Hu et al [30]. have adopted research methods from other disciplines to examine the national spatial data. Here, the systems theory was used to understand the wetland production-living-ecological complex system and its synergies. Meanwhile, Gu et al [31]. used the system dynamics theory to predict the urbanization rate of China in the next 50 years, while Yi et al [32]. also adopted the same theory in municipal territorial spatial planning. Hence, system dynamics can be a suitable approach in predicting future development changes and solving complex nonlinear system problems, using scenario simulations and models that combine qualitative and quantitative data on various system levels and their interactions.

Generally, existing studies on the coordination of production-living-ecological functions have been performed from a systematic perspective. However, these have been primarily focused on the internal mechanisms of human-land relationship coordination [33,34], and strategies to promote the coordination, balance and sustainable development of geographical environment and human well-being [35,36]. Meanwhile, research particularly focused on the coordination between human and "production-living-ecological" functions have remained lacking, in which only few studies have attempted to combine only two of the "production-living-ecological" functions, and urban ecology, production, living functions. The projections of the interrelationships between the "production-life-ecology" functions of the future city are limited, which does not provide recommendations for the future development of the city and alerts for risk avoidance. Moreover, urban ecology, production, living functions, and their correlation to the people have not been extensively explored under the system dynamics framework. This study uses the theoretical framework of the human-earth system. It also applies the system dynamics theory [37] using a holistic-systems thinking approach to develop the complex "production-living-ecological" system, consisted of the urban production, living, and ecology systems. Using Changsha City, China, as a case study area, we employ system dynamics modeling to project the future development of production, living, and ecological functions within the city. By exploring the external characterization of the system layer by layer to the internal structure of the system, the operation mechanism of the integrated state of the urban human-land coupling and coordination relationship is obtained.[38]. It is expected that this study may provide valuable insights and recommendations to improve the spatial governance of Changsha and develop a theoretical framework for new urbanization approaches.

## Material and methods

### Study area and data sources

**Fig 1:**
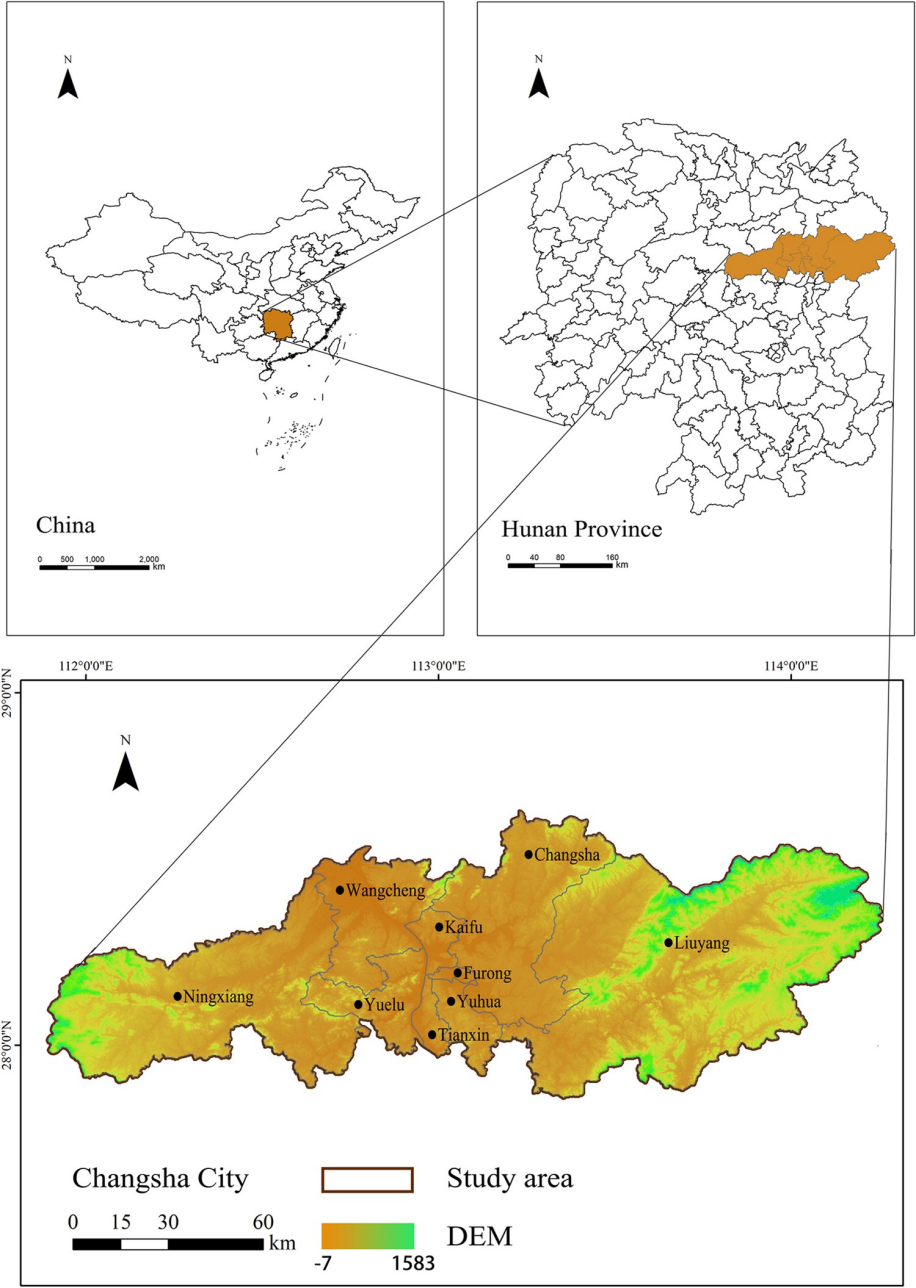
Location of Changsha, the study area in the middle of China.

Changsha City is in the northeastern portion of Hunan Province in China (Fig 1, created using ARCGIS 10.2), covers an area of 11,819 km2. It is an important node city of the central urban agglomeration of the Yangtze River and the Yangtze River Economic Belt. It includes six districts, with one county and two county-level cities, such as Yuelu, Yuhua, Furong, Tianxin, Kaifu, and Wangcheng District and Changsha County, as well as Liuyang City and Ningxiang City so it has an outstanding locational advantage. In 2020, it had a population of approximately 10 million, increasing by 42.71% over the last decade. In 2020 gross domestic product (GDP) in 2020 was 1214.252 billion yuan, signifying a 4% increase relative to its previous year [39,40], well above the provincial average. Additionally, Changsha serves as a pivotal grain production center in China and a testing ground for the comprehensive reform of the "two-oriented society" [41]. Furthermore, its historical and cultural significance further underscores its research value. For a special human geographic unit such as Changsha, as the core growth pole of economic development in Hunan Province, it is extremely important to implement qualitative and quantitative research on the prediction of the future development of its urban "production-life-ecology". In China, there are many Chinese cities that are similar to Changsha in terms of geographic location, resources, and economic development, similar geographic units to Changsha City also include Wuhan and Suzhou, especially Wuhan, which shares many similarities with Changsha in terms of geographic location, ecological environment, and economic development, and many scholars have also focused on the development of Wuhan’s "production-life-ecology" space. For example, scholars used a system dynamics model to study the coordination of the "production-life-ecology" space in Wuhan and Suzhou [42,43]. Based on the previous studies, the importance of Changsha’s geographic location and its rapid economic and social development make it feasible research area for this study, and the study of Changsha can provide a reference for the development of Chinese cities.

The model data were obtained from the Statistical Yearbook of Hunan Province and the Statistical Yearbook of Changsha City for the period 2010–2019.

## Methods

Qualitative and quantitative analyses were used to analyze the dynamic characteristics of the production-living-ecology system, including its structure and subsystems and their interactions, and predict future development changes in the subsystems.

### Qualitative analysis of the urban production-living-ecology complex system model

**Fig 2:**
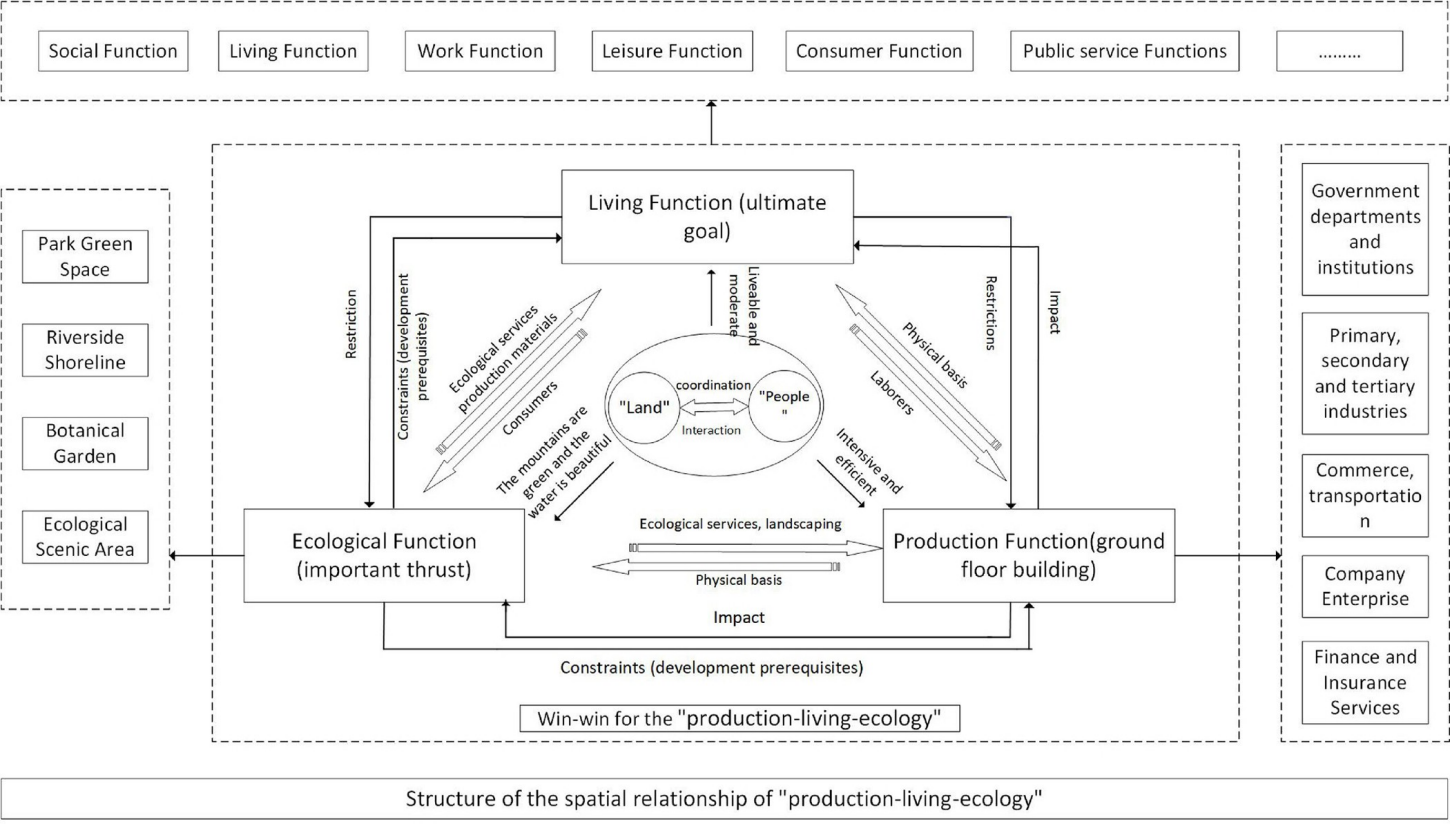
Function relationship structure of the production-living-ecology system.

A composite system is a high-level system generated from the coupling of two or more systems, in which the original system is considered as one of its subsystems. Here, various interactions between the elements occur [44,45]. Specifically, a coupled coordination not only represents the state of the system, but also its imposed role on the system. Coordination in the former refers to the harmonious relationship between various elements, including their cooperation, complementarity, and synergy that allows the system to maintaining an optimum overall effect or function [46]. Using this qualitive relationship, Fig 2 shows the relationship between human-land coordination and prime urban functions development.

Production function involves the utilization of land as the means of labor to directly generate a wide array of products and services, such as the primary, secondary, and tertiary industries to provide residents with the supplies and services needed for production and life including the financial and insurance services industry to provide as a subdivision of the production function. In addition, the life function refers to the various spatial bearings, material and spiritual security functions provided by the land during the process of human survival and development. The life function refers to the various spatial carrying, material and spiritual protection functions provided by the land in the process of human survival and development. It includes elements like leisure, which contributes to people’s well-being by offering relaxation and enhancing their overall quality of life. Additionally, it involves the consumption function, facilitating shopping and consumption for city residents. Both leisure and consumption functions are subdivisions of the life function. Lastly, the ecological function relates to the ecosystems and ecological processes that sustain the natural conditions necessary for human survival and well-being. This encompasses ecological scenic areas, parks, and green spaces that maintain a vital ecological environment for both life and work [47,48].

1. "Causal cycle" characteristics of the urban "production-living-ecological" system

The urban system is composed of multiple elements that interact and overlap each other, forming complex causal relationships. This causal relationship is the basic law of urban systems and considered as the basic unit for analyzing urban complex systems. The economist Gonna Murdar stated that urban systems involved a process of continuous evolution, in which technological, social, economic, and cultural factors, among the few, contribute to this as interrelating, and mutually influencing and causal factors that form a vicious cycle with cumulative effects [49]. As cities, production, living, and ecological factors also interact with each other in the "production-living-ecological" system, a vicious cycle may occur in cases where one factor is mismanaged. For instance, an uncoordinated development of urban life and production factors may lead to a destruction of ecological factors, which may consequently affect production development. Furthermore, a poor environment condition may lead to lesser business investments. Poor development and utilization of land use affects the potential overall quality of life of the population, owing to reduced happiness and increased disease incidence. Consequently, this lowers the capacity for improving and protecting ecological spaces, as financial and material resource investments are also reduced, forming a vicious circle. Hence, analyzing the urban complex system in consideration with the mutual causality between urban subsystems based on the current urban development conditions is necessary.

2. Dynamics and Sustainable Development Characteristics of the Urban " production-living-ecological " System

Cities are inherently influenced by internal and external factors and are constantly moving and changing. In the process of development, negative impacts such as environmental pollution, pressure on urban infrastructure, and housing constraints arise from the disruption of urban system functions, in which it consequently leads to economic, social, and ecological imbalances and threaten sustainable urban development. Impacts such as these are also simultaneously coupled with the increasing intensity of anthropogenic activities, which further obscures the boundaries between the interacting subsystems [50,51]. Sustainable development of cities requires a coordinated development of the urban production, living, and ecological elements, ensuring that immediate and long-term interests are accounted for and the degree of development of each element and their interaction with the other elements are understood to ensure synergy between these elements within the entire system.

3. People and place synergy in the urban "production-living-ecological" system

The urban "production-living-ecological" system consists of various synergies among its subsystems and elements. It is essentially considered as a human-land relationship system as the "production-living-ecological" comprise the environmental elements, while the various activities occurring comprise the "human elements". Here, the overall stability is dependent of the level of coordination between and among these elements. For instance, a carrying capacity exists for environmental elements, in which a potential irreversible collapse may occur as a result of intensive human activities. Hence, a balance between these elements requires a harmonious coexistence and interaction between human and land [52]. This relationship also comprises the various functions of the subsystems within the human-land system [53], in which the production, living, and ecological spatial subsystems form a feedback system that is essential to understanding how the complex urban system can be optimally managed. In addition, understanding its historical evolution process is also required as it may serve as basis for potential urban development strategies, accounting for the driving mechanism of change through time. This is also to ensure that an objective approach is taken and potential personal interests in urban development are eliminated to ensure the achievement of sustainable urban development.

4. Integrity and openness of the urban "production-living-ecological" system

A city is the center of human life and business production. It is an open system as exchanges of internal elements between its internal and external areas are constantly and continuously occurring. In this process, unfavorable exchange conditions inevitably arise that makes the entire urban system function in both order and disorder. An open system tends to be disorderly as collisions such as urban political territory and urban identity issues occur and disrupt its equilibrium [54]. Therefore, a disorderly state can be transformed into an orderly state by coordinating the various elements, particularly the production, living, and ecology elements. A higher degree of decrease in disorder of the system requires a higher degree of coordination [54]. In this study, the urban area of Changsha was also considered as an open system, in which its internal "production-living-ecological" functions was attempted to be coordinated to ensure that an optimal orderly state is achieved, in addition to maintaining an efficient material and energy exchange between its internal and external urban areas.

5. Self-adaptability and self-organization of the urban "production-living-ecological" system

Composite systems are a combination of natural and man-made systems, in which it has the ability to both self-organize and regulate while also being externally regulated and managed through the introduction of various methods. Urban composite systems possess a characteristic of these two systems, where it can self-organize as a result of various human activities [55,56]. Hence, a coordinated and balanced human production activity with ecological buffers ensure the capability of the system to self-organize and regulate. However, when human interests such as those activities involving the prioritization of higher economic gains without consideration of the resource carrying capacity, urban economic destabilization and ecological imbalance ensue [57], which consequently affects the self-adaptive capacity of the urban system. To avoid this, urban systems must be allowed to recover and self-organize using appropriate land planning and management.

Through the qualitative analysis mentioned above, from a systemic point of view, in the process of urban development, cities have the characteristics of a systemic "cause and effect cycle", dynamics and sustainable development, synergy between people and land, wholeness and openness, as well as self-adaptation and self-organization, which clearly determine the fundamental problems of the city. To gain a clear understanding of fundamental urban issues, we must initiate our analysis from within the city’s internal structure, while duly considering all constituent elements of the "people" and "land" relationship.

### Quantitative analysis of the urban complex production-living-ecology system

As an urban system is complex with multiple components and dynamic interconnections, a single qualitative analysis cannot solely determine its behavioral and functional characteristics. This study explores the coupling and coordination of the "production-living-ecological" system in Changsha to determine the dynamics of the production-living-ecology system by constructing a composite system. It also aims to predict the changes of variables such as GDP in the model using quantitative analysis, as changes in GDP affect fiscal revenues that determines the amount of resource allocation for urban green space areas, including pertinent ecological indicators.

1. Causal feedback flow chart

The mechanism of interaction between the living and production subsystem involves provision of the necessary material materials for the living by the production, while the living subsystem concurrently provides the necessary labor for the production, in addition to acting as consumers as well. Hence, a satisfactory living can consequently improve the production efficiency.

Meanwhile, the production subsystem produces solid, gas, and liquid wastes, that are excreted from production areas to the ecological environment. This causes ecological environmental pollution. However, improving production benefits may also provide the necessary economic support to further protect the physical environment and its ecological functions, because only with a developed economy and increased financial revenues, people’s investment in ecological protection increases relatively. Among other things, a developed economy can also lead to a higher environmental awareness among its people.

The mechanism of interaction between the ecological and living subsystems involves the provision of a good living environment for the people, resulting in an improved quality of life and happiness. Consequently, the former also affects the latter through generation of domestic garbage that reduces the quality of living function.

**Fig 3:**
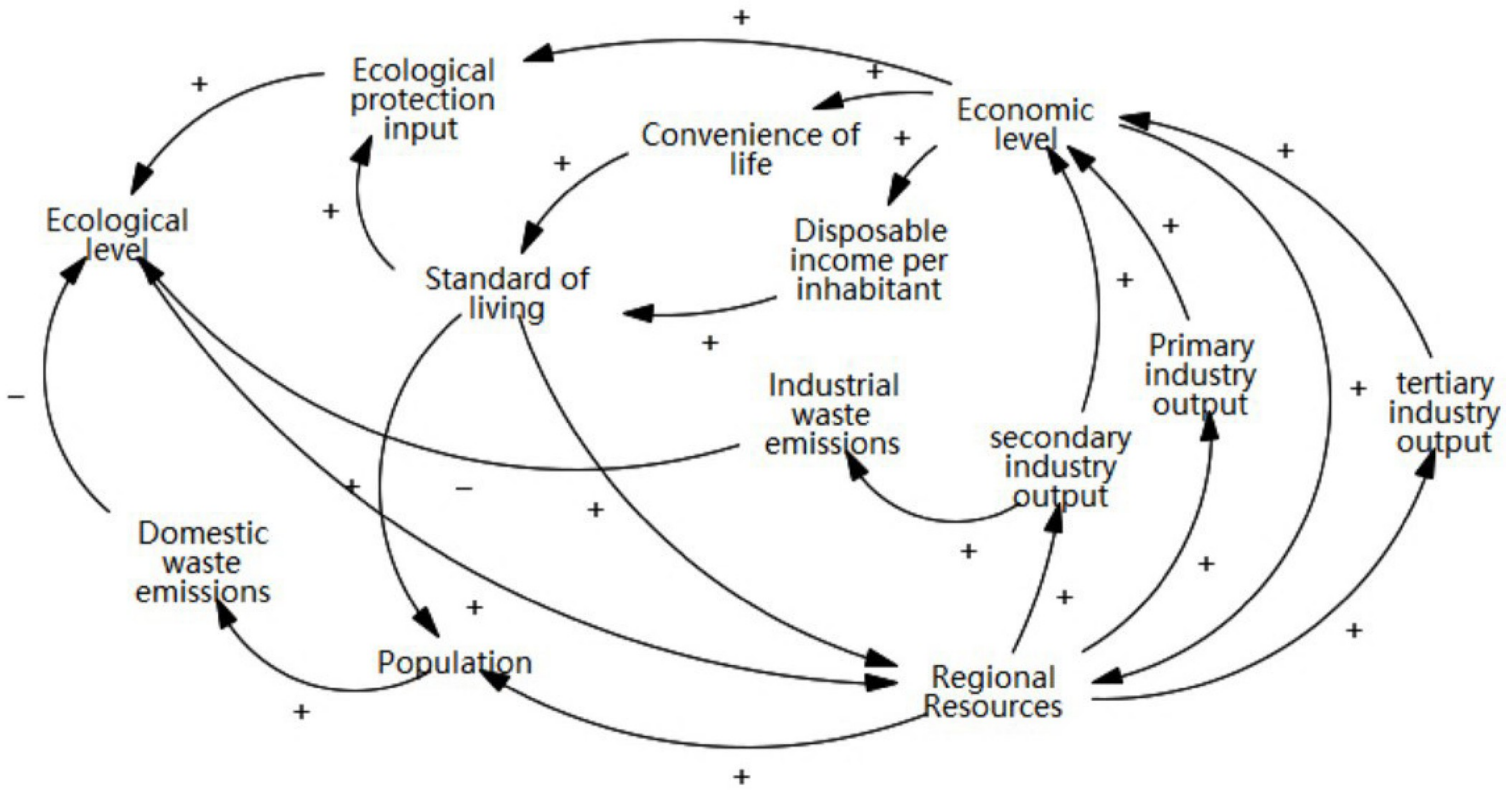
Causal feedback loop of the production-living-ecological system.

Based on these feedback systems, the Vensim PLE software was used to analyze the causal feedback flow diagram of the of production, living, and ecological subsystems in Changsha, as shown in Fig 3.

2. System structure flow diagram

**Fig 4:**
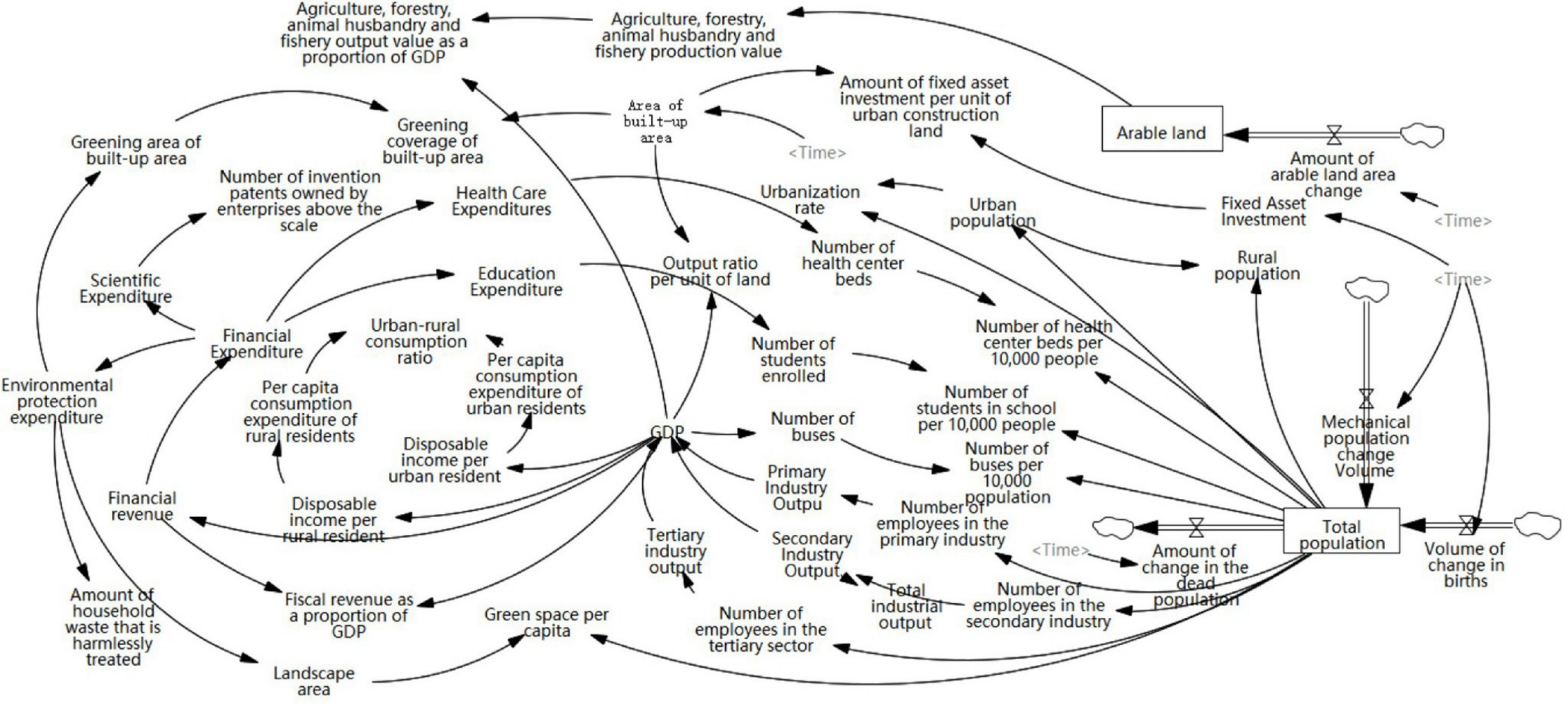
Structural flow diagram of the production-living-ecological system in Changsha City.

The causal feedback loop diagram depicts the fundamental linkages and causal interconnections between subsystems, whereas system dynamics flow diagrams are needed for a more in-depth analysis of the interactions of the system’s components and the forecasting of the system’s future. Referring to previous studies, the variables in the model below were selected. The Vensim PLE software-based production-living-ecological system flow diagram for the city is displayed (Fig 4). Based on this, the framework for the production-living-ecology complex system dynamics model was built.

3. System dynamics equation

The urban production-living-ecology system is characterized by a complex and dynamic cause-effect cycle, integrity and openness, self-organization, and self-adaptive system dynamics. These complexities have a significant impact on development and human-earth synergy. Hence, general mathematical methods were not sufficient to provide quantitative and accurate descriptions and analysis. For instance, the "production-living-ecological" coupling formula can only examine the level of reciprocal coupling among urban systems, but cannot accurately forecast future system evolution. Instead, we employed a system dynamics model, which linearly analyses GDP, total population, landscaped area, and arable land as the influencing factors was linearly analyzed to provide a more accurate representation of the complex relationship between the subsystems. Here, that the state variables of production, living and ecosystem were interrelated and mutually exclusive and were derived from advanced artificial determination. Based on a large body of literature, the correlation between similar measures of "production-life-ecology" in Chinese cities has been confirmed by numerous studies. The system dynamics equations as detailed in (Table 1), describe the quantitative interrelationships between variables in the structural flow diagram of a system using historical data fitting variables of Changsha, the current data was used to predict and analyze future conditions in 2035.

**Table 1:** Equation of production-living-ecological complex system in Changsha City.

Type of function	Indicators	Formula
**Function for production**	Primary Industry Output	-0.1573*Number of employees in the primary industry + 149.14
	Secondary Industry Output	0.0114*Number of employees in the secondary industry + 107.9
	Tertiary industry output	0.013*Number of employees in the tertiary sector + 153.34
	GDP	Primary Industry Output + Secondary Industry Output + Tertiary industry output
	Financial Expenditure	0.6352*Financial revenue + 3e+06
	Financial revenue	0.188*GDP-5e+06
	Fiscal revenue as a proportion of GDP	Financial revenue/GDP
	Disposable income per rural resident	3.2054*GDP—4177.3
	Disposable income per urban resident	4.5768*GDP + 1680.5
	Arable land	Arable land + Amount of arable land area change
	Area of built-up area	Area of built-up area + Amount of change in built-up area
	Output ratio per unit of land	Area of built-up area/GDP
	Agriculture, forestry, animal husbandry and fishery output value as a proportion of GDP	Agriculture, forestry, animal husbandry and fishery production value/GDP
	Agriculture, forestry, animal husbandry and fishery production value	(-10.475) *(Arable land*Arable land) + 5701.4*Arable land—775222
	Amount of fixed asset investment per unit of urban construction land	Fixed Asset Investment/Area of built-up area
	Fixed Asset Investment	WITH LOOKUP ([(0, 0)—(10, 10)], (2010, 3192.57), (2011, 3510.24), (2012, 4011.96), (2013, 4593.39), (2014, 5435.75), (2015, 6363.29), (2016, 6693.32), (2017, 7567.77), (2018, 8087.33), (2019, 8735.4))
	Number of invention patents owned by enterprises above the scale	0.052*Scientific Expenditure- 4370.6
	Total population	Volume of change in births + Mechanical population change Volume-Amount of change in the dead population
**Function for living**	Urban population	0.6894*Total population + 370.39
	Rural population	Total population-Urban population
	Urbanization rate	Rural population/Total population
	Number of employees in the primary industry	-0.1831*Total population + 240.5
	Number of employees in the secondary industry	2.9579*Total population + 284.94
	Number of employees in the tertiary sector	0.4446*Total population—130.54
	Per capita consumption expenditure of rural residents	0.9252*Disposable income per rural resident—6424
	Per capita consumption expenditure of urban residents	0.7774*Disposable income per urban resident—2457
	Urban-rural consumption ratio	Per capita consumption expenditure of urban residents/Per capita consumption expenditure of rural residents
	Scientific Expenditure	5e-09*(Financial Expenditure*Financial Expenditure)- 0.0728*Financial Expenditure + 447697
	Health Care Expenditures	0.0537*Financial Expenditure- 16790
	Education Expenditure	0.144*Financial Expenditure + 77966
	Number of health center beds	0.0705*Health Care Expenditures + 30598
	Number of students enrolled	300000*Education Expenditure+ 110.05
	Number of health center beds per 10,000 people	Number of health center beds/Total population
	Number of students in school per 10,000 people	Number of students enrolled/Total population
	Number of buses	0.0002*(GDP*GDP) - 1.5993*GDP + 7200.9
	Number of buses per 10,000 population	Number of buses/Total population
**Ecology Function**	Amount of household waste that is harmlessly treated	0.0003*Environmental protection expenditure + 113.27
	Environmental protection expenditure	0.0482*Financial Expenditure- 109700
	Greening coverage of built-up area	Greening area of built-up area/Area of built-up area
	Landscape area	0.0115*Environmental protection expenditure + 8796.4

*Note*: The equation for the Number of health center beds per 10,000 people contains two variables, the Number of health center beds and the Total population is obtained by dividing the Number of health center beds by the Total population. The Number of health center beds is determined by calculating the linear relationship between historical statistics of the Health Care Expenditures in Changsha City and our own data of the Number of health center beds over the years.4. Testing of the model.

The results of the simulation part of the system dynamics simulation were compared and analyzed with the existing historical data to test the reliability of the model. However, owing to the complexity of the model and large number of variables, this study focused on the historical verification of the changes in the total population, built-up area, arable land area, GDP, and landscaped area of Changsha City. The errors between the simulated data and the actual data were mostly within 10%, and the model exhibited a relatively reliable result (Table 2).

**Table 2:** Model history test results.

Projects		2010	2011	2012	2013	2014
**Total population**	Actual value	704.07	709.07	714.66	722.14	731.15
	Simulated value	704.07	706.02	717.31	724.48	735.28
	Relative Error	0	0.4	0.3	0.3	0.5
	Simulated value	4440.32	5320.01	6211.23	7354.61	7989.55
	Relative Error	0	5.3	2.9	2.8	2.1
**Arable Land Area**	Actual value	276.79	275.65	274.89	274.15	273.36
	Simulated value	276.79	267.22	264.13	263.32	265.03
	Relative Error	0	3.0	3.9	3.9	3.0
**Built-up area**	Actual value	272.39	306.39	315.81	325.51	336.25
	Simulated value	272.39	297.67	304.29	306.37	347.98
	Relative Error	0	2.8	3.6	5.8	3.4
**Landscape area**	Actual value	9857	10235	10729	11206	11813
	Simulated value	9857	10102	10324	10612	12015
	Relative Error	0	1.2	3.7	5.3	1.7

## Results

### Qualitative analysis of the complex "production-living-ecological" system structure in Changsha City

**Fig 5:**
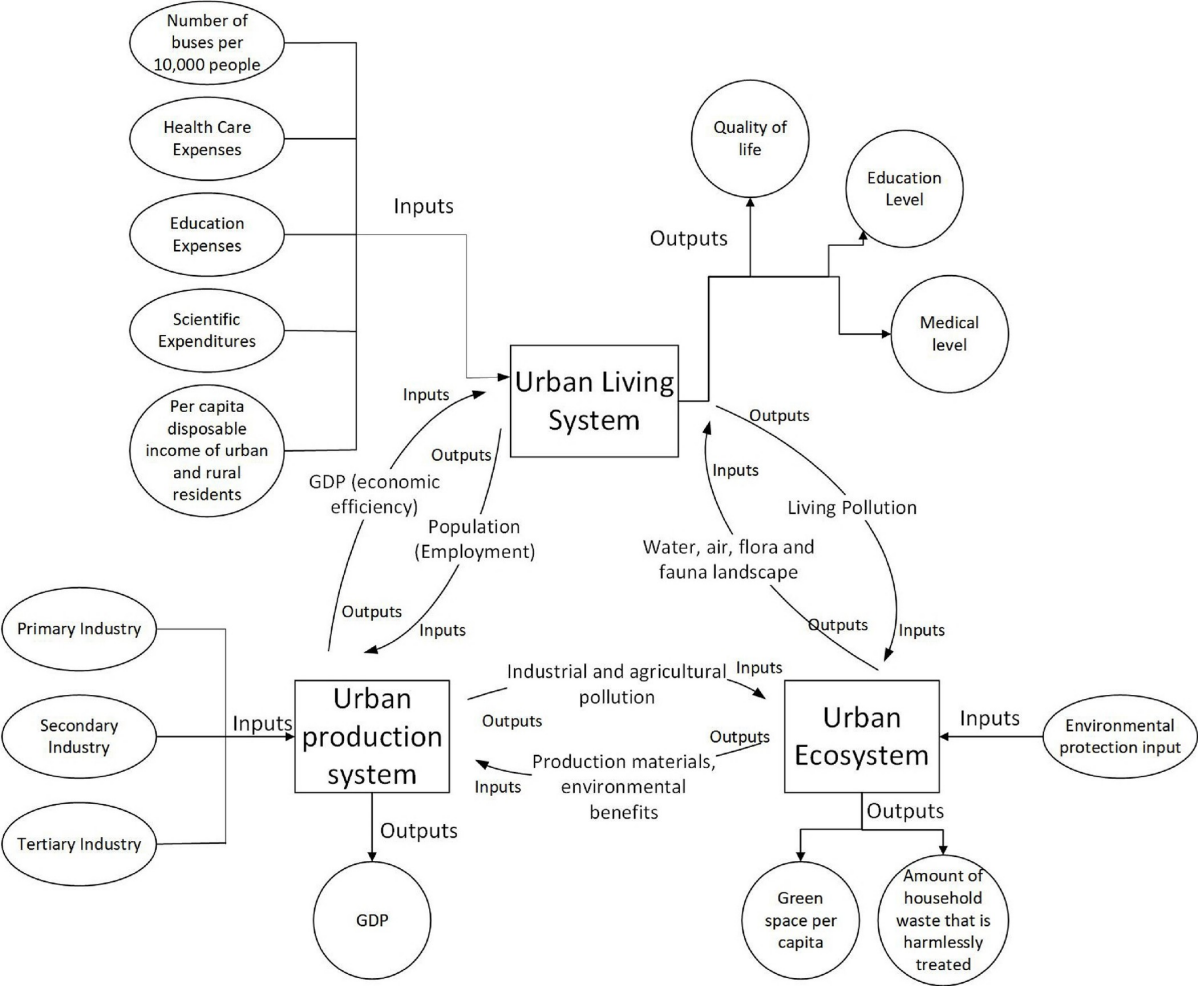
Structure of production-living-ecological complex system.

The concept of the "production-living-ecological" system was proposed in this study based on land use functions, derived from the "element-structure-function" in the systems theory, as the system function is dependent on the structure of the system. Each subsystem of the complex production-living-ecology system was developed and operated to obtain a coordinated structure. Fig 5 presents the internal structure analysis diagram used in this study.

#### Productive subsystem of Changsha City

The production subsystem is composed of industrial, commercial, and other economic activity spaces in the city. It provides the necessary material conditions for urban development, and it has a non-negligible influence on the other subsystems in the model. It encompasses all the primary economic production function of the country, including both direct and indirect economic productions. The former refers to all areas that directly generate goods and services such as agricultural lands, whereas the latter refers to all areas used for intermediate transport and housing of these goods and services such as urban construction areas. As Changsha City is mainly an industrial area, it is primarily composed of areas under indirect production [58,59].

Human activities are fundamentally driven by addressing human needs, making them integral to the indirect production process. In recent years, the production space in Changsha City has been mainly shaped by the secondary and tertiary industries. The development of the secondary and tertiary industries relies on the development of high-quality talents and basic labor force, as well as tourism consumption due to increase in population. These factors are closely related to living space and ecological space. Consequently, the internal structure of the production subsystem should be appropriately coordinated with the ecological and living subsystems to provide a better material basis for a more optimum living function balanced with green ecological function.

#### Living subsystem of Changsha City

The living function refers to the sum of various areas for daily human activities. According to Wang [60], living function cover six aspects based on the conduct of these daily human activities: living function, working function, leisure function, consumption function, public service function, and social function. Furthermore, the living subsystem often overlap with the production and ecological subsystems, as these are areas designed to meet basic living needs, which cannot be easily reduced or transformed and tends to restrict the development of the other subsystems. Generally, the ultimate goal of urban development is to satisfy these basic needs to a high level, Livability, comfort, and convenience form the core aspects of living space. However, when the resident population experiences rapid growth alongside a shortage of public service resources, it results in resource limitations, traffic congestion, and inadequate public services.

#### Consequently, addressing these challenges must take precedence in the coordinated development of Changsha City. Ecological subsystem of Changsha City

The ecological refers to the functional characteristics of the physical environment, in which it provides the resources and inputs used for human activities such as organic matter and raw materials for food and material production, and ecological services such as biodiversity, climate regulation and mitigation, natural disaster protection, pest control and environmental purification, for the overall human well-being [61]. Hence, the ecological environment is key to protecting urban growth [62]. The ecological subsystem operates as an open system that is closely connected linked with the surrounding production and living subsystems, facilitating exchange of materials within and between regions. For example, the industrial pollution produced from the production function and the domestic garbage pollution by the living function will negatively impact ecological function. When the ecological function is compromised, healthy working and living environments is not conducive to the development of the city. To achieve an optimum value of the ecological function, the ecological subsystem must have an appropriate internal and external structure that is balanced with the living and ecological subsystems to allow symbiotic interactions and meet the development needs of Changsha City [52].

### Coupling and coordination quantitative analysis of the "production-living-ecological" space system in Changsha City

**Fig 6:**
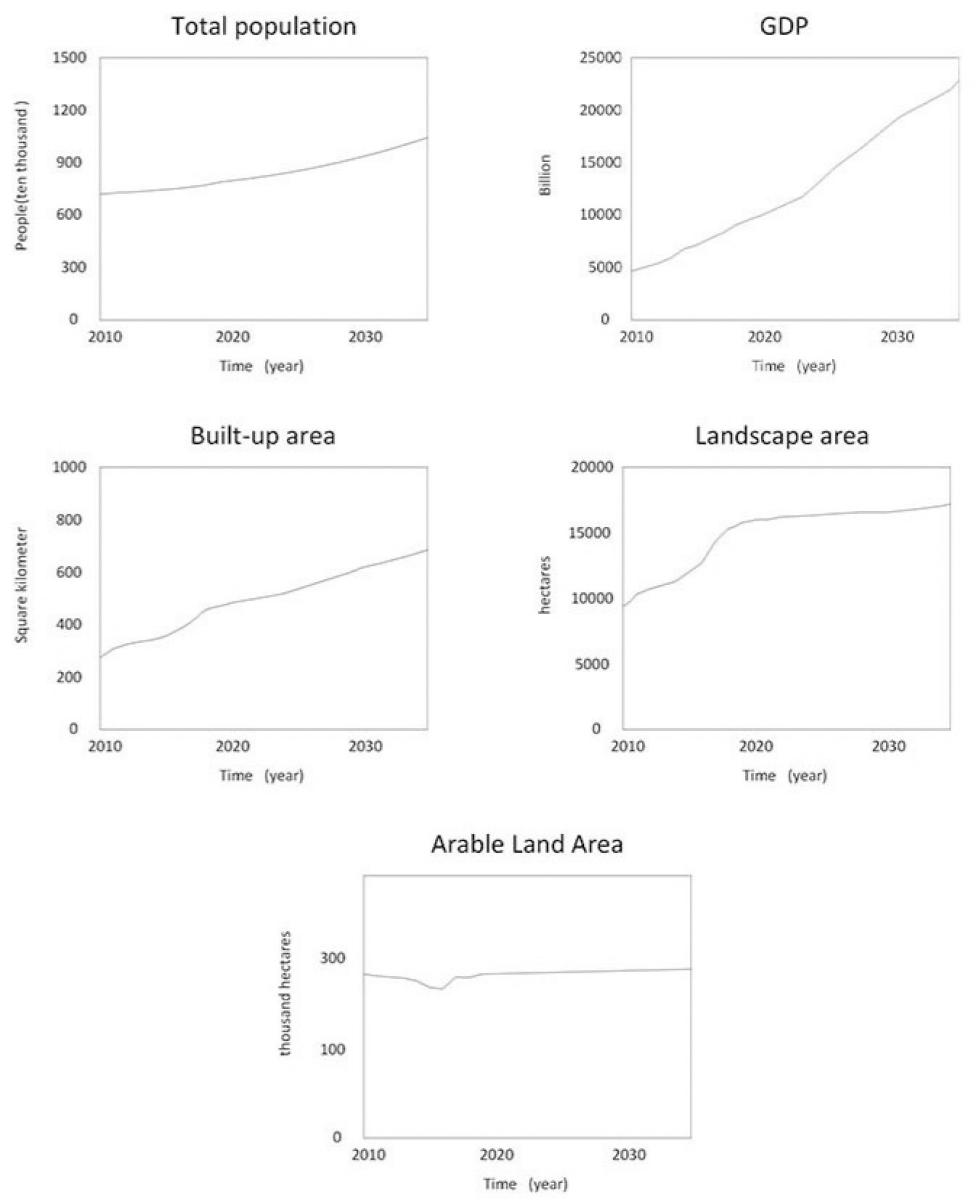
15-year projection of the production-living-ecological urban complex system in Changsha.

According to the Changsha Statistical Yearbook, the total population of Changsha City in 2010 was 7,040,700, and the total GDP was 444,032 billion yuan; the per capita urban disposable income in 2010 was 23,347 yuan; the per capita rural disposable income in 2010 was 10,640 yuan. The arable land area in 2010 was 276.79 thousand ha, and the built-up area was 27239 ha. This study measures the development changes of the production-living-ecological system of Changsha City in the next 15 years based on the conditions from 2010–2019(Fig 6). Here, employment in the primary industry, secondary industry, and tertiary industry were derived from the total population, in which its corresponding output values were expected to reach 3,693.66 billion yuan in GDP in 2035.The GDP and living standard also gradually increased, in which the total population was positively correlated with the total GDP. Meanwhile, the economic development will promote the increase of population, as more public service infrastructures are constructed, which maintains the residence of local population in the city and increases the foreign population. Population growth also promotes the development of urban production function, such as accelerated urbanization resulted in the encroachment of the rural population into the urban areas, which would consequently increase the labor force and lower the labor cost. Similarly, it also increased consumption, which revitalized economic production and consumption. This reflects the relationship between population development and economic development. Here, gradual upward trends were observed. The expansion of built-up area is impacted by the rise of GDP. while gradually increases in the built-up areas were also observed along with the development of production space. Built-up areas were predicted to reach 70821.8 ha in 2035.Growth in built-up area as a result of economic progress and population growth However, because Changsha’s total area is limited, and ecological function takes a portion of it, the built-up area reaches a plateau, and the rate of growth gradually slows.

As shown in Fig 6, the economic development particularly increased green spaces, which improved environmental protection. On the one hand, people are more aware of environmental protection and have higher requirements for their living and working environment; on the other hand, economic development brings more investment in environmental protection. The reciprocal relationship between productive development and ecological protection is reflected here. Future scenarios indicate that Changsha City will continue to comply with the management requirements of the "three zones and three lines", implementing appropriate arable land protection measures. With this, slight changes in the arable land areas in Changsha City may be observed. It was also observed that the overall development of Changsha City required the preservation of ecological security and basic food security to have healthy and stable economic development, in which the production, living, and ecological functions the intensity of human activities must be maintained below the carrying capacity of the geographic environment. Long-term urban planning projects will shape the urban environment for decades, not just a single or two year cycles. Obtaining accurate urban growth projections is necessary to create a scientific city plan. The above predictions for Changsha’s progress over the next 15 years may provide urban planners, government agencies, and other stakeholders with a scientific basis.

## Discussion

### Contribution of system dynamics theory to the study of urban system development

As a composite system, the "production-living-ecological" system of Changsha City was found to have a significant causal cycle, in which the internal factors have interrelated influence on each other, that may potentially form a beneficial or vicious cycle. The characteristic of the cycle depends on the degree of coupling and coordination of the systems [63]. For example, in the context of coupled production and living systems, Changsha’s population growth within a reasonable range will bring labor force and consumption power to the economic development. However, if population development exceeds the carrying capacity of Changsha’s resources, it will lead to some social problems such as traffic congestion, housing tension, urban villages and so on. Therefore, the development of Changsha City needs to be approached from a systemic point of view, from all aspects within the subsystems, to explore in depth the problems encountered in its development. With the dynamic and open characteristic of Changsha City, efficient communication with external resources is required to achieve efficient coupling of resources within the system to derive its benefits and ensure synergy and sustainability between the people and land [62]. With this, it provides new insights on urban planning approaches that incorporates a systemic perspective and eliminates traditional methods to urban planning.

### System dynamics modelling for urban system collapse risk avoidance

While single analytical framework generally places the issue of "urban riskification" in a static system of categorization, along with globalization and post-industrialization, different types of urban risks have become intertwined, overlapping and compounding each other in a systemic way [64]. The case study of Changsha City demonstrates that cities are coupled within the system and interacts altogether, suggesting that the primary, secondary and tertiary industries, urban built-up area, and landscaped area are tightly linked. The entire system may be disrupted when one of these variables are overactive and uncontrolled. For instance, when human activities exceed the carrying capacity of the physical environment such as when there is uncontrolled population growth, the urban ecology is threatened, owing to the amount of waste disposed in the environment. Further, an oversupply of labor and a shortage of labor products also threatens the production function, which creates social problems, owing to unemployment. When the ecological environment is damaged, the comfort of urban living functions decreases, population out flows, and subsequently reduces labor force decreases consumption power. This, in turn, impacts the city’s productive functions, leading to urban decline.

### The generalizability of urban "production-life-ecology" prediction models in the process of urban development

a) Scientific prediction of urban land use change can avoid the potential risk of urban expansion and other development modes to a certain extent [65]. The model in this paper aims to provide a certain theoretical basis for assessing future urban land use risks.

b) The innovation of energy technology. The enhancement of production function can provide sufficient financial support for the innovation of energy technology, which is a kind of mutual feedback mechanism. As the convenience and comfort of residents’ life increases and the ecological environment improves, the city can attract high-quality talents and improve the efficiency of energy technology innovation [66].

c)The guidance of the central government’s policy has a strong determining effect on the change of the city’s "production-life-ecology" function, and it is necessary to weigh and consider all the factors when optimizing the city’s planning strategy using the model presented in this paper. d) The central government’s policy essential to alter the city’s "production-life-ecology" function. The optimization of urban planning strategies using this model must be evaluated against the guidance of the central government’s policies and the specificity of the model itself [67].

d)Reform and redesign of agricultural production methods, which is a significant aspect of the city’s production function, is closely related to the innovation of energy technology, and indicates the continuous improvement of the model. The reform and redesign of agricultural production methods, which is closely related to the innovation of energy technology, indicates the continuous improvement of the model. The reform and redesign of the production methods of agriculture. Similarly, agricultural production is an important aspect of the productive function of the city, closely related to energy technology innovation, and indicative of the continuous improvement of the urban production-life-ecology model. The results of this study are similar to the findings of the study of the "Literature of the City" [68–70].

Globally, regarding the "production-life-ecology" development of cities, substantial studies have focused on the efficient development and utilization of land functions, established a comprehensive indicator system with 29 indicators and formulated a set of comprehensive assessment methods [71]. In addition, regarding the development of urban "production-life-ecology", previous studies revealed the importance of coordinating the promotion of the integration of production and urbanization with carbon emission reduction for the construction of a green economic system and in-depth participation in global environmental governance [72].

### Limitations and prospects

Although Changsha City is comparatively more developed than other regions in central China, a coupled and coordinated development within the city is insufficient. Rather, the focus must be on urban development at a regional level. Secondly, the analysis model needs to be further optimized by selecting a higher number of model variables and designing an improved causal feedback path. Data accessibility was also limited, which affected data refinement. It is recommended that more accurate and comprehensive data on urban production, living, and ecology are incorporated. Moreover, the comprehensive and accurate data increases the generalizability and scientific validity of models. Furthermore, variable selection and indicators must be more defined to establish a more systematic. System models at different scales with subtle differences in variable selection. The system dynamics model emphasizes the coordination of subsystems and the synthesis of the larger system. At both township and city scales, the coupling and coordination of internal subsystems are essential for the city system to operate efficiently. However, there are differences in the internal subsystems and elements. For example, the township production function mainly refers to the agricultural, whereas cities focus on the indirect production of products derived from the primary sector. A more representative urban "human-land" relationship and situational simulation will be pursued in subsequent studies. Additionally, exploring micro-levels such as cities and towns may yield refined results.

## Conclusions

The aim of this study was to investigate the mechanisms and coupling coordination between the "production-living-ecological" system of a city such as Changsha to determine the interaction among production, life, and ecological space subsystems. A qualitative analysis of the system dynamics was conducted to understand the characteristics and structure of the system, while a system dynamics model of the production-living-ecology complex system was constructed using the Vensim PLE software to determine the internal structure of the complex urban system. It was found that the overall trend of population, GDP, and built-up areas showed an upward trend. A clear correlation among the three factors was also found, in which mutual correlations among the "production-living-ecological" system had a significant influence on urban development and quality of life. Furthermore, this study showed that the system dynamics model can be highly applicable in investigating the coordination of urban production, living, and ecological functions. In the system dynamics model, each variable is interconnected with each other, and none of them exists independently. Our findings conclude that the production, living, and ecological functions of Changsha City are interacting with each other. The model in this paper predicts the changes of Changsha City in the next fifteen years can provide a scientific basis for the government policy makers and urban planners to plan the development of the production-living-ecology functions in the future. It is recommended that more accurate data are used to build a more representative model. This study provides a new basis for decision makers in improving and ensuring sustainable urban development.
